# Quorum-sensing regulation in staphylococci—an overview

**DOI:** 10.3389/fmicb.2015.01174

**Published:** 2015-10-27

**Authors:** Katherine Y. Le, Michael Otto

**Affiliations:** ^1^Pathogen Molecular Genetics Section, Laboratory of Bacteriology, National Institute of Allergy and Infectious Diseases, National Institutes of HealthBethesda, MD, USA; ^2^Division of Hospital Internal Medicine, Department of Medicine, Mayo Clinic College of MedicineRochester, MN, USA

**Keywords:** *Staphylococcus aureus*, *Staphylococcus epidermidis*, quorum-sensing, toxins, biofilm, Agr, LuxS

## Abstract

Staphylococci are frequent human commensals and some species can cause disease. *Staphylococcus aureus* in particular is a dangerous human pathogen. In staphylococci, the ability to sense the bacterial cell density, or quorum, and to respond with genetic adaptations is due to one main system, which is called accessory gene regulator (Agr). The extracellular signal of Agr is a post-translationally modified peptide containing a thiolactone structure. Under conditions of high cell density, Agr is responsible for the increased expression of many toxins and degradative exoenzymes, and decreased expression of several colonization factors. This regulation is important for the timing of virulence factor expression during infection and the development of acute disease, while low activity of Agr is associated with chronic staphylococcal infections, such as those involving biofilm formation. Accordingly, drugs inhibiting Agr are being evaluated for their capacity to control acute forms of *S. aureus* infection.

## Introduction

Staphylococci, including *Staphylococcus aureus* and the coagulase-negative staphylococci, the most common of which is *Staphylococcus epidermidis*, have been implicated as causative agents in a variety of human infections, including infections involving the skin and soft tissue, the blood stream, the respiratory system, the skeletal system, as well as infections involving implanted medical devices. Due to their ability to aptly regulate a wide armamentarium of virulence factors, including toxins, degradative enzymes, antimicrobial resistance genes, and immune evasion mechanisms, staphylococci not only are able to successfully secure infectious niches in a wide range of organ systems, but are also successful pathogens in both acute and chronic human infections (Lowy, 1998; Otto, 2009).

Among the regulatory mechanisms that ensure timely adaptation of staphylococcal physiology to the environment, quorum-sensing stands out as one of the most intensely studied and probably most important mechanisms for the control of pathogenesis. Quorum-sensing is population density-dependent and environment-dependent gene regulation that occurs through cell-cell communication (Waters and Bassler, 2005). The role of two regulatory systems in staphylococci, the accessory gene regulator (Agr) system and the LuxS system, are discussed herein. Agr is considered the prototype quorum-sensing regulator system in Gram-positive bacteria (Kleerebezem et al., 1997), while the role of LuxS as a quorum-sensing system in staphylococci is less certain.

## The Agr system

**Figure 1:**
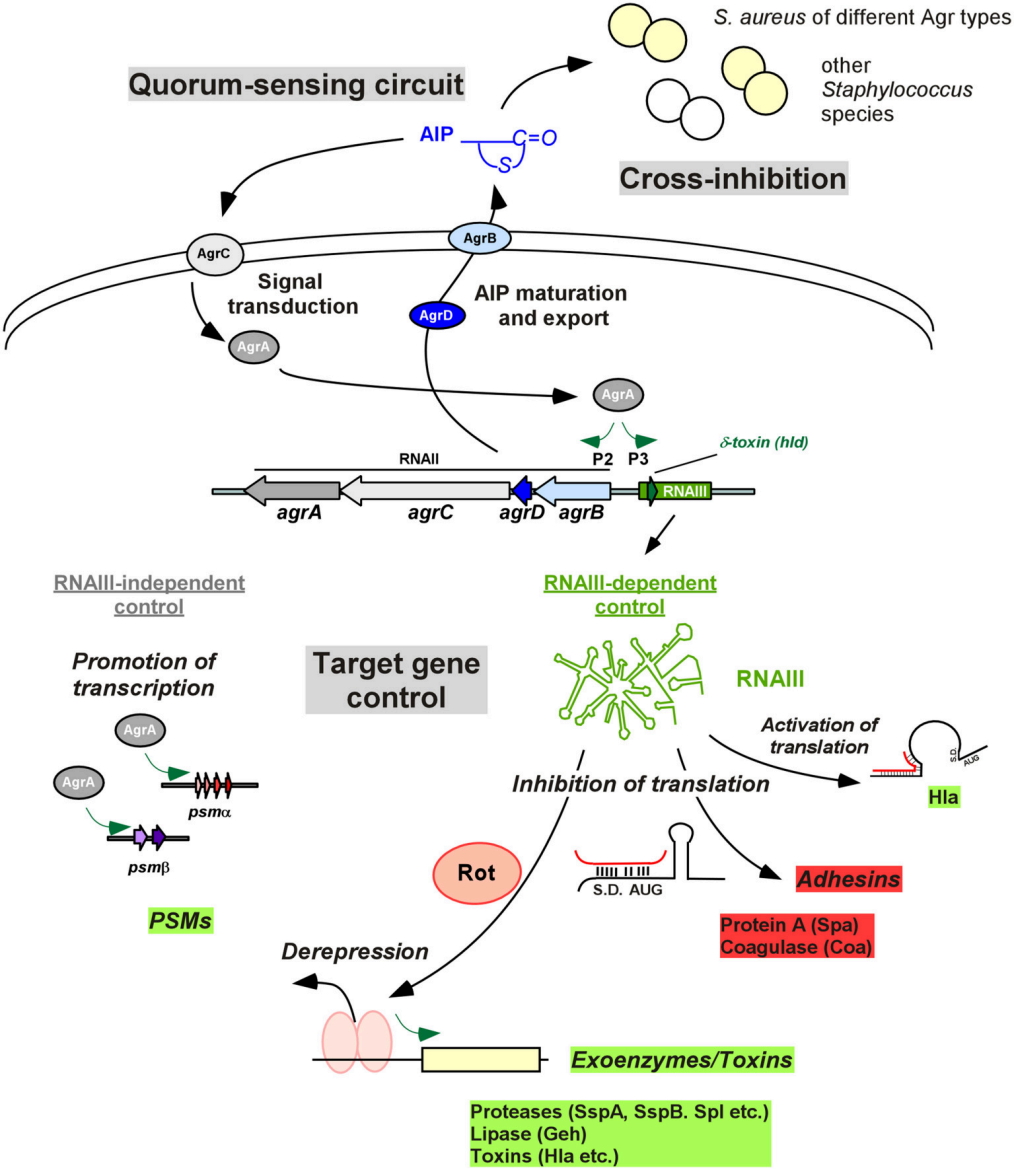
**Agr control in ***Staphylococcus*****. The quorum-sensing circuit is shown at the top left. The AIP signal is produced from the AgrD precursor by AgrB maturation and export. At a certain threshold concentration, AIP activates the AgrC-AgrA two-component system and phosphorylated AgrA activates transcription from the P2 promoter, resulting in auto-feedback regulation. An important feature of Agr is group specificity, resulting in cross-inhibition, i.e., inhibition of Agr activity, in strains belonging to other Agr specificity groups and other species (top right). Target gene control is shown at the bottom. Most Agr targets are regulated via RNAIII, whose transcription is increased by AgrA, via the P3 promoter. RNAIII also contains the gene for delta-toxin (*hld*). RNAIII controls target genes by base pairing with 5′UTRs, in most cases inhibiting translation. This is used for direct inhibition of Agr-inhibited target genes such as protein A, while inhibition of translation of the repressor Rot leads to de-repression of transcription of many of the classical Agr target toxins, such as alpha-toxin. AgrA also increases transcription of the *psm*α and *psm*β operons, encoding PSM peptides, in an RNAIII-independent mode of Agr target gene regulation. S.D., Shine/Dalgarno sequence; AUG, start codon of controlled gene.

The *agr* locus is 3.5 kb in size and consists of two divergent transcriptional units, RNAII and RNAIII, whose transcription is driven by the P2 and P3 promoters, respectively (Peng et al., 1988; Kornblum et al., 1990; Figure 1). The RNAII locus contains four genes, *agrB, agrD, agrC and agrA* (Kornblum et al., 1990; Figure 1). The *agrD* transcript encodes a peptide precursor of the extracellular quorum signal of Agr, called autoinducing peptide (AIP) (Ji et al., 1995). The AIP in its mature form is 7–9 amino acids long and contains a characteristic thiolactone (or in *Staphylococcus intermedius*, lactone) ring between the centrally located cysteine and the C terminus (Ji et al., 1997, 2005; Otto et al., 1998; Novick and Geisinger, 2008). The *agrB* gene product is a transmembrane endopeptidase that is responsible for the introduction of the thiolactone modification, C-terminal cleavage, and export of the AIP (Saenz et al., 2000; Zhang et al., 2002; Zhang and Ji, 2004); in the extracellular milieu the AIP receives final trimming by the type I signal peptidase SspB (Kavanaugh et al., 2007). The *agrC* and *agrA* genes encode a two-component signal transduction system involving a histidine kinase sensor AgrC, a transmembrane protein that is phosphorylated upon the binding of AIP (Lina et al., 1998), and its associated response regulator AgrA (Novick et al., 1995; Queck et al., 2008). Upon being activated by AgrC-dependent phosphorylation, AgrA binds to the P2 promoter region for RNAII and the P3 promoter region for RNAIII (Koenig et al., 2004), as well as the promoters controlling expression of the PSMα and PSMβ peptides (Novick et al., 1995; Queck et al., 2008; Figure 1).

Although *agr* is generally conserved within the staphylococci, variations in the sequences of *agrB, agrC, agrD* lead to the production of AIPs with varied signaling specificities, allowing for activation of self and cross-inhibition of Agr groups of non-self (Ji et al., 1997; Otto et al., 2001; Olson et al., 2014), a phenomenon that might be reflective of evolutionary selective pressures (Novick and Geisinger, 2008). Besides the AIP, Agr can also be activated by a variety of other regulators, such as SarA (Heinrichs et al., 1996) or SrrAB (Yarwood et al., 2001), and environmental factors such as glucose concentration or pH (Regassa et al., 1992).

### RNAIII-dependent gene regulation

RNAIII is the intracellular effector molecule of the Agr system responsible for the control of Agr targets (Novick et al., 1993). It is also a messenger RNA, containing the *hld* gene for delta-toxin (or delta-hemolysin) (Janzon et al., 1989). The primary mechanism by which RNAIII controls target gene expression is by antisense base pairing with 5′ untranslated regions (5′ UTRs), forming RNA duplexes. Except for in the case of alpha-toxin, in which RNAIII may also act as a post-transcriptional activator (Morfeldt et al., 1995), RNAIII usually blocks translation (Boisset et al., 2007). By this direct mechanism, RNAIII inhibits the production of a series of predominantly surface proteins, such as protein A and others. Furthermore, RNAIII blocks translation of the repressor of toxin (Rot) protein, which belongs to the staphylococcal accessory regulator (Sar) transcriptional regulator family (McNamara et al., 2000; Saïd-Salim et al., 2003; Boisset et al., 2007). Rot binds to the promoter region of many exoproteins and toxins, blocking their transcription. These mechanisms allow for prompt density-dependent up-regulation of enterotoxins, alpha-toxin, leukocidins, degradative exoenzymes and down-regulation of surface proteins. Moreover, genome-wide analyses of gene expression indicate that in addition to the control of virulence determinants, Agr control also comprises a series of metabolic targets (Dunman et al., 2001; Yao et al., 2006; Cheung et al., 2011). These general physiological adaptations may assist to adapt bacterial physiology to the changed requirements during infection.

### RNAIII-independent gene regulation

While RNAIII was long believed to represent the only mechanism by which Agr controls target genes, it was found more recently that the response regulator AgrA not only binds to the P2 and P3 promoters, but also directly up-regulates transcription of the phenol-soluble modulin *psm*α and *psm*β operons by binding to the respective promoter sequences (Queck et al., 2008). The phenol-soluble modulins (PSMs) are a family of staphylococcal peptide toxins that also include the delta-toxin (Cheung et al., 2014a). As the delta-toxin gene is embedded within RNAIII, it has been speculated that evolution of RNAIII around the delta-toxin gene connected an evolutionarily more ancient regulatory network consisting of AgrA and possibly quorum-sensing control of *psm* genes to the control of further virulence determinants via RNAIII and Rot (Queck et al., 2008).

### The impact of Agr on acute infection and toxicity

In *S. aureus*, the up-regulation of virulence factors by Agr is necessary for disease progression in several animal models of acute infection, including infective endocarditis (Cheung et al., 1994), skin and soft tissue infections (Wright et al., 2005; Cheung et al., 2011), pneumonia (Heyer et al., 2002; Bubeck Wardenburg et al., 2007a; Montgomery et al., 2010), and septic arthritis and osteomyelitis (Abdelnour et al., 1993; Gillaspy et al., 1995). Conversely, down-regulation by Agr of PSMs and microbial surface components has been implicated in enhanced biofilm formation and bacterial colonization of indwelling medical devices (see below) (Vuong et al., 2000, 2004; Wang et al., 2011; Periasamy et al., 2012). Moreover, Agr dysfunction is correlated with persistent *S. aureus* bacteremia (Fowler et al., 2004). In general, the cell density/Agr-dependent up-regulation of toxins and degradative exoenzymes on the one hand and the down-regulation of surface components on the other is supposed to reflect the differential temporal requirements for specific virulence determinants during the course of a bacterial infection: in the beginning of an infection, low cell density and consequential low expression of Agr results in increased production of surface components required for the initial colonization of tissues. Once this is established, bacteria grow to higher cell densities, requiring additional food sources and increased protection from host defenses, which is accomplished by Agr-dependent up-regulation of degradative exoenzymes and toxins. Notably, this timing also ascertains that production of toxins, many of which are pro-inflammatory, is delayed until the growing bacterial colonies can deal with attacks by host defenses, which likely explains the findings particularly related to the role of Agr and Agr-controlled factors during *S. aureus* bacteremia (Fowler et al., 2004; Cheung et al., 2014b). Finally, Agr-controlled expression of virulence determinants is energy-consuming and there are several recent reports indicating that this needs to be balanced with the expression of antibiotic resistance in antibiotic-resistant strains (Joo et al., 2010; Rudkin et al., 2014). Specifically, subinhibitory concentrations of antibiotics have been shown to increase Agr expression (Joo et al., 2010; Paulander et al., 2013; Rudkin et al., 2014), imposing a fitness cost, which has been speculated to drive the observed formation of Agr-dysfunctional mutants in strains isolated from hospital infections (Traber et al., 2008; Paulander et al., 2013).

### Agr-controlled virulence determinants

One of the most prominent Agr-regulated toxins is alpha-toxin (alpha-hemolysin, Hla), which is encoded by the *hla* locus. It is a 319 amino acid beta-barrel pore-forming toxin that binds to the disintegrin and metalloprotease 10 (ADAM10) receptor on the host cell membrane (Wilke and Bubeck Wardenburg, 2010; Berube and Bubeck Wardenburg, 2013). Hla has been implicated in multiple staphylococcal infections in humans. Isogenic *hla* mutants consistently demonstrated reduced disease severity when compared to the wild-type strain when examined in animal models involving pneumonia (Bubeck Wardenburg et al., 2007a,b), skin and soft tissue infection (Kennedy et al., 2010; Kobayashi et al., 2011), and endovascular infection (Powers et al., 2012).

The bi-component leukocidins constitute another family of generally Agr-controlled pore-forming toxins, which includes Panton-Valentine Leukocidin (PVL), γ-hemolysin, LukDE, and LukGH (LukAB) (Queck et al., 2008; Cheung et al., 2011; Alonzo and Torres, 2014). While PVL is now believed to play a more limited role in severe staphylococcal infections than previously thought (Otto, 2011), LukED has emerged as an important factor controlling *S. aureus* disease development (Gravet et al., 1998; Alonzo et al., 2012). LukED causes lysis of phagocytic cells by targeting the CCR5 chemokine receptor on lymphocytes, macrophages and dendritic cells (Alonzo et al., 2013). Furthermore, LukGH (LukAB) appears to play a key role in *S. aureus* pathogenesis, as it is the only *S. aureus* toxin in addition to the PSMα peptides that contributes to lysis after phagocytosis (DuMont et al., 2013).

PSMs are a family of peptide toxins (Cheung et al., 2014a) that stand out among Agr-controlled virulence factors as the only ones that are under direct control by AgrA (Queck et al., 2008), emphasizing a likely central role in staphylococcal physiology that is underlined by their role in the non-infectious lifestyle of staphylococcal commensals (Cheung et al., 2014a). Among the PSMs, those encoded in the *psm*α locus of *S. aureus*, most notably PSMα3, are strongly pro-inflammatory and lytic to a variety of cell types, including neutrophils, macrophages, osteoblasts, and erythrocytes (Wang et al., 2007; Rasigade et al., 2013). As a consequence, PSMα peptides have a strong impact on acute types of *S. aureus* infection, such as skin and soft tissue infections, sepsis, and osteomyelitis (Wang et al., 2007; Cassat et al., 2013).

Many secreted enzymes are under regulation by Agr. Several proteases in particular are very strongly Agr-controlled and in addition to representing virulence factors in their own right (by contributing to the degradation of tissue and host defense proteins such as antimicrobial peptides), they can shape the composition of the pathogen secretome and thus its pathogenic properties (Cheung et al., 2011; Kolar et al., 2013).

Among the generally Agr-down-regulated surface proteins, protein A (Spa) stands out, due to its multifactorial impact on pathogenesis in addition to the fact that it appears to be consistently Agr-controlled (Cheung et al., 2011). In contrast, the general dogma of negative control by Agr of other surface proteins does not hold true in all strains (Cheung et al., 2011). Protein A has been implicated in infection models involving multiple organ systems, including pneumonia (Heyer et al., 2002; Bubeck Wardenburg et al., 2007a; Montgomery et al., 2010), septic arthritis (Palmqvist et al., 2002), and bloodstream infection (Date et al., 2014). It has a role in triggering pro-inflammatory mediators in the lung parenchyma through activation of the TNF-α receptor (TNFR1) (Gómez et al., 2004), and also has the capacity to inactivate the host humoral immune response upon release from the staphylococcal envelope (Becker et al., 2014) by binding the Fcγ domain of IgG to block F_c_ receptor-mediated opsonophagocytosis (Peterson et al., 1977), and also by binding to the F_ab_ domain of V_H_3-clan IgM and triggering apoptosis (Goodyear et al., 2006; Pauli et al., 2014).

### Agr regulation of biofilms and biofilm-associated infection

Biofilm formation is thought to occur through several stages: (1) attachment of microorganisms to abiotic or host matrix protein-coated surfaces and aggregation into multicellular structures, (2) proliferation/maturation, and (3) detachment/dispersal (Otto, 2008). Initially speculated to lead to biofilm dispersal, based on the observation that *agr* mutants showed extended biofilm formation in both *S. aureus* and *S. epidermidis* (Vuong et al., 2000, 2003, 2004), the detailed mechanisms by which Agr impacts biofilm development have recently become clear.

Agr controls proteases, which *in vitro* impact biofilm extension by degrading protein components of the biofilm matrix (Lauderdale et al., 2009). However, the *in vivo* relevance of proteases in staphylococcal biofilm formation is debatable (Otto, 2013). The only biofilm-structuring and dispersal mechanism whose relevance has been confirmed *in vivo*, and in experiments using biological fluids, is based on PSM surfactant function (Wang et al., 2011; Periasamy et al., 2012; Dastgheyb et al., 2015). In a process that is largely independent of the mode of biofilm formation, i.e., the composition of the biofilm matrix, PSMs structure biofilms by forming channels and lead to the dispersal of cells from the biofilm. This process leads to the dissemination of biofilm-associated infection *in vivo* and is responsible for the extensive biofilm formation that is observed under conditions of low Agr activity, such as in synovial fluid during joint infection (Otto, 2013; Dastgheyb et al., 2015). Thus, the impact of Agr on biofilm-associated infection is divergent: Agr is necessary for biofilm structuring and the dissemination of biofilm infection, but dysfunction of Agr leads to enhanced biofilm formation, which may be advantageous for the bacteria under those conditions. Accordingly, strains with a dysfunctional Agr system are often isolated from infections on indwelling devices (Vuong et al., 2004; Traber et al., 2008) and colonizing strains (Shopsin et al., 2008). These strains have lost the capacity to disseminate in the patient's body as well as to other individuals (Shopsin et al., 2010).

## LuxS

Described in *Vibrio* spp., the regulatory effect of *luxS* was discovered in the context of bioluminescence regulation, and since has been recognized as a widely utilized quorum-sensing system among bacteria, prompting speculation of its role in interspecies communication (Bassler et al., 1997; Bassler, 2002). The LuxS system employs an autoinducer called AI-2, which is a furanosyl borate diester molecule (Chen et al., 2002). Several phenotypes, such as capsule synthesis, biofilm formation, antibiotic susceptibility, and virulence, have been linked to AI-2 regulation in *S. aureus* (Zhao et al., 2010; Yu et al., 2012; Xue et al., 2013). Similarly, in *S. epidermidis*, the *luxS* gene was found to impact the expression of a series of genes, including biofilm exopolysaccharide biosynthesis (Li et al., 2008). Furthermore, a *luxS* mutant demonstrated increased virulence in a catheter-related murine infection model (Xu et al., 2006). This finding was later corroborated under both *in vitro* and *in vivo* experimental conditions in *S. aureus*, where *luxS* exerted control of biofilm growth through the *icaR* locus (Yu et al., 2012). However, the role of LuxS as a quorum-sensing regulatory system in staphylococci remains under debate. In fact, it has been argued that the impact of a *luxS* deletion on the observed phenotypes in staphylococci may be due only to its primary role in metabolism, where it is an integral part of the S-adenosyl methionine cycle (Winzer et al., 2002; Doherty et al., 2006).

## Therapeutic approaches targeting staphylococcal quorum-sensing

There has been continued interest in targeting Agr for the development of anti-staphylococcal drugs (Khan et al., 2015). One such effort exploits the cross-inhibiting activity of Agr AIPs and includes altering the AIPs through amino acid substitution, relocation of the amino acid side chains, removal of the exocyclic tail, and AIP polymerization, in order to enhance the stability of the AIPs and make them more resistant to proteolysis (Mayville et al., 1999; Affas et al., 2001; Lyon et al., 2002; Melamed Yerushalmi et al., 2013; Tal-Gan et al., 2013; Khan et al., 2015). Furthermore, monoclonal antibodies against a specific AIP were effective in suppressing Agr expression in an Agr strain of a different Agr specificity group and protected mice against a lethal *S. aureus* challenge of that strain (Park et al., 2007). Finally, there have been promising recent approaches targeting the AgrA response regulator by a small molecule, savirin (Sully et al., 2014). Notably, in that study the virulence-suppressing activity did not result in any development of resistance, which is an often claimed but infrequently tested supposed advantage of anti-virulence drugs.

However, a noticeable problem with targeting Agr remains the dichotomy of Agr control. Due to the phenotype of biofilm enhancement in *agr* mutants, which has been confirmed using a cross-inhibiting AIP (Vuong et al., 2004), the impact of Agr-inhibiting drugs such as those derived from cross-inhibiting AIPs, on chronic and biofilm-associated forms of staphylococcal infection would be counterproductive. Due to the predominant involvement of coagulase-negative staphylococci in biofilm infections (Otto, 2004), Agr blockers would certainly not be used for infections by those strains. Nevertheless, Agr inhibitors may form valuable drugs for the inhibition of acute toxicity in many *S. aureus* infections, possibly to be given together with conventional antibiotics (Khan et al., 2015).

## Conclusion

Quorum-sensing regulation in staphylococci is mainly due to the Agr locus, which regulates a wide variety of virulence determinants in addition to metabolic genes. Accordingly, there is a strong impact of Agr on many types of staphylococcal disease. In acute types of disease, Agr generally enhances pathogenesis by increasing expression of aggressive virulence determinants such as toxins and degradative exoenzymes. In contrast, Agr has a more complicated role during chronic infections, as mutants in Agr show increased biofilm formation, but decreased potential to disseminate, in addition to being correlated with enhanced success during persistent bacteremia. Compared to Agr, the role of LuxS in staphylococcal quorum-sensing is less well understood.

Clearly, there is renewed recent interest in targeting Agr for drug development, which appears a valid option for acute disease types. However, going forward, the often divergent roles of Agr in various types of staphylococcal infection need to be understood better to establish a solid scientific basis underscoring the applicability of quorum-sensing blockers for the treatment of staphylococcal disease.

### Conflict of interest statement

The authors declare that the research was conducted in the absence of any commercial or financial relationships that could be construed as a potential conflict of interest.
